# Risk factors for suicidal ideations and suicide attempts among medical students

**DOI:** 10.1192/j.eurpsy.2024.1642

**Published:** 2024-08-27

**Authors:** M. Turki, W. Abid, O. Khardani, N. Halouani, M. Bakallah, S. Ellouze, J. Aloulou

**Affiliations:** ^1^Psychiatry B, Hedi chaker university hospital, Sfax, Tunisia

## Abstract

**Introduction:**

Medical students are a high-risk population for having suicidal thoughts and behaviors. However, few studies have been fulfilled on this subject.

**Objectives:**

The objective of this study was to provide a systematic overview of risk factors for suicidal ideations (SI) and suicide attempts (SA) among medical students.

**Methods:**

We set out to summarize the literature on the MEDLINE (via PUBMED) and Science Direct databases, regarding risk factors for SI and SA in medical students, using the key words : « medical student» ; « suicide attempt» ; « suicidal ideation », « risk ».

**Results:**

Recent studies showed that poor mental health outcomes including depression, anxiety, burnout, comorbid mental illness, and stress presented the strongest risk for SI among medical students. In addition, SI was statistically significantly associated with alcohol use, Tobacco consumption, personal history of suicide attempt, female gender and poor social support.

On the other hand, SA were significantly associated with the presence of a long-term illness, anxiety and depression. Conversely, stress, female gender, and alcohol use were not significant risk factors for SA among medical students.

**Conclusions:**

Medical students face a number of personal, environmental, and academic challenges that may put them at risk for SI and SA. Additional research on individual risk factors is needed to construct effective suicide prevention programs in medical schools.

**Disclosure of Interest:**

None Declared

